# From Resuscitation to Rehabilitation: The Post-Intensive Care Syndrome Continuum in Sepsis Care

**DOI:** 10.3390/jcm14238374

**Published:** 2025-11-26

**Authors:** Matthew Sherman, Perry Lim, Tariq Cheema, Briana DiSilvio, Perry Tiberio

**Affiliations:** 1 Department of Internal Medicine, Allegheny Health Network, Pittsburgh, PA 15212, USA; 2 Drexel University College of Medicine, Philadelphia, PA 19104, USA; pyl24@drexel.edu; 3 Division of Pulmonary and Critical Care, Allegheny Health Network, Pittsburgh, PA 15212, USAbriana.disilvio@ahn.org (B.D.); perry.tiberio@ahn.org (P.T.)

**Keywords:** sepsis, septic shock, post-intensive care syndrome, cognitive dysfunction, physical impairment, psychological disease

## Abstract

Sepsis and septic shock affect nearly 49 million people worldwide each year. Although advances in early recognition and evidence-based management have improved survival, many patients experience long-term cognitive, physical, and psychological impairments collectively known as post-intensive care syndrome (PICS). These sequelae often extend to families and caregivers (PICS-F), resulting in lasting declines in quality of life. Recovery from sepsis represents a continuum that begins during intensive care and extends into survivorship. Decisions regarding analgesia, sedation, delirium prevention, mobilization, and family engagement shape this recovery trajectory. The ABCDEF bundle provides an evidence-based framework to mitigate these long-term effects through structured approaches to pain control, paired awakening and breathing trials, targeted sedation, early mobility, and family involvement. This narrative review synthesizes current evidence on PICS in sepsis and septic shock and examines how implementation of the ABCDEF bundle across the continuum of care can reduce the incidence and severity of post-ICU impairments. Reframing sepsis survivorship as an ongoing process rather than an endpoint underscores the need for critical care practices that promote not only survival but also restoration of function, cognition, and quality of life.

## 1. Introduction

Sepsis, a condition characterized by a profound and dysregulated systemic response to infection, affects nearly 40% of patients admitted to intensive care units (ICU) [1,2,3]. Advances in early recognition and evidence-based management have led to substantial reductions in mortality over the past two decades [1,4,5,6,7]. Yet, as survival has improved, the focus of critical care has expanded beyond acute resuscitation to encompass the long-term sequelae of critical illness. Many sepsis survivors experience new or worsening cognitive, physical, and psychological impairments—collectively termed post-intensive care syndrome (PICS)—that persist long after hospital discharge [8,9,10].

Recovery from sepsis thus represents a continuum that begins during ICU care and extends into survivorship. Within this continuum, the ABCDEF (A2F) bundle—which integrates pain and sedation management, spontaneous awakening and breathing trials, delirium monitoring, early mobility, and family engagement—provides a structured framework to improve both short- and long-term outcomes. In this review, we discuss current evidence on PICS among patients with sepsis and septic shock, emphasizing how implementation of the ABCDEF bundle across the continuum of care may mitigate long-term cognitive, physical, and psychological impairments and promote meaningful recovery after sepsis.

## 2. Methods

A targeted narrative review using PubMed and the Cochrane Library from January 2000 to August 2025 was performed. Search terms included ‘sepsis’, ‘septic shock’, ‘post-intensive care syndrome’ and were combined with ‘cognitive’, ‘cognitive dysfunction’, ‘physical’, ‘physical dysfunction’, ‘mental health’, ‘mental disorder’, ‘anxiety’, ‘depression’, ‘post-traumatic stress disorder’, and ‘psychological’. Consensus meetings and statements and large cohort studies were prioritized. Landmark studies and significant topic-specific contributions preceding 2000 were retained as foundational background.

### 2.1. The Recovery Trajectory: From Resuscitation to Rehabilitation

Sepsis and septic shock affect nearly 49 million people annually with mortality ranging from 15 to 50% [11,12,13,14,15,16]. Continued efforts to improve outcomes have led to several updates to the Surviving Sepsis Campaign, most recently published in 2021 [17]. These guidelines emphasize key interventions such as appropriate volume resuscitation, early antibiotic administration, vasoactive therapy with advanced hemodynamic monitoring, and early ICU admission [17]. While these strategies have contributed to reduced mortality, patients with these syndromes continue to experience prolonged ICU stays and complex treatment regimens [2]. As advances in sepsis management have reduced mortality, there has been increased recognition of the complicated recovery experience of these patients.

**Figure 1:**
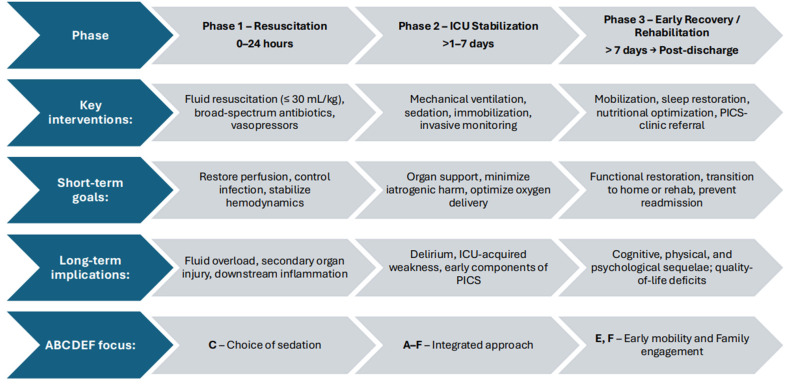
Proposed Continuum from Resuscitation.

Recovery from sepsis begins at the moment of resuscitation (Figure 1). Early resuscitation quality remains a critical determinant of outcomes. Delays in antibiotic initiation have consistently demonstrated stepwise increases in mortality, with each hour of delay after hypotension associated with a significant rise in mortality [18,19]. Similarly, excessive fluid resuscitation has been linked to higher rates of pulmonary edema, acute kidney injury, and prolonged mechanical ventilation, particularly in patients with cardiac or renal dysfunction [20,21,22]. Conversely, adherence to early, balanced resuscitation principles—timely antibiotics, cautious volume expansion, and hemodynamic-guided vasopressor use—has been associated with shorter ICU stays, improved organ recovery, and decreased long-term cognitive and physical impairment [17]. Each decision—from antibiotic timing and volume resuscitation to sedative choice and depth of sedation—has lasting effects on patients’ long-term recovery trajectories. Recognizing this as a longitudinal process—one in which early ICU decisions influence outcomes in the form of post-intensive care syndrome—helps reframe sepsis management as a continuum from survival to recovery.

### 2.2. Post-Intensive Care Syndrome (PICS)

**Figure 2:**
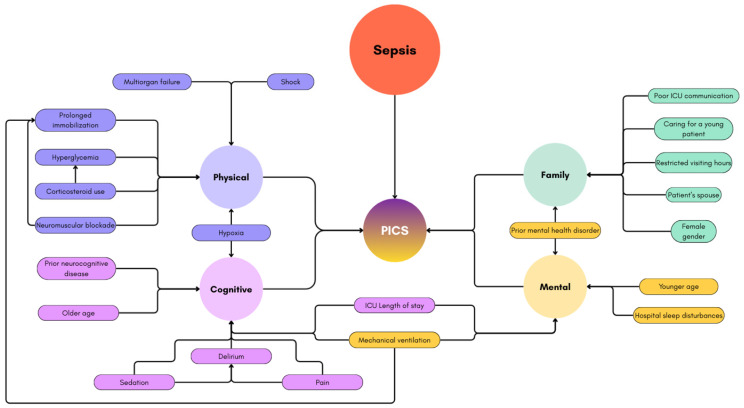
Risk Factors for the Development of PICS in Patients with Sepsis and Septic Shock.

Defined in 2010 by the Society of Critical Care Medicine, post-intensive care syndrome (PICS) encompasses new or worsening impairments in physical, cognitive, or mental health that arise following survival of a critical illness [8]. The overall prevalence of PICS in survivors of septic shock is high, and the reported prevalence depends on the study population and timing of assessment. A recent meta-analysis including more than 10,000 ICU survivors reported a PICS prevalence of 54–62% up to two years after hospital discharge [9]. In patients with septic shock, a 2025 multicenter study demonstrated that 85% met criteria for PICS at discharge, with 45% continuing to experience symptoms at one year [10]. The following sections summarize current evidence on PICS as it relates to sepsis and septic shock, focusing on prevalence, risk factors (Figure 2), underlying mechanisms, clinical manifestations, and long-term outcomes.

### 2.3. PICS-Cognitive Dysfunction

Sepsis and septic shock syndromes are characterized by profound neuroinflammatory responses that disrupt blood–brain barrier integrity, impair cerebral perfusion, and alter neurotransmission. These mechanisms contribute to the development of delirium and subsequent cognitive dysfunction, key components of PICS [10]. Delirium occurs in up to 70% of septic ICU patients, and retrospective studies report significant cognitive impairment in nearly 30% of survivors one year after critical illness [10,23,24]. Risk factors include older age, pre-existing neurocognitive disorders such as Alzheimer’s dementia, and prolonged exposure to sedatives, analgesics, or mechanical ventilation. The presence and duration of ICU delirium constitute the strongest predictors of long-term cognitive dysfunction [23,25]. These impairments can persist for several years, with one study showing cognitive impairment among survivors of severe sepsis for up to 8 years after acute illness [26].

### 2.4. PICS-Physical Impairment

Sepsis and septic shock trigger a cascade of systemic and cellular responses that contribute to profound skeletal muscle injury and physical impairment. Pro-inflammatory cytokines, microvascular dysfunction, mitochondrial injury, and catabolic hormone signaling lead to critical illness myopathy and polyneuropathy, the primary mechanisms underlying post-ICU weakness [27,28,29]. Among survivors of septic shock, physical dysfunction is reported in approximately 25–65% at one year [9,10,30]. Clinical manifestations include generalized and respiratory muscle weakness, impaired mobility, difficulty regaining muscle mass, incontinence, sexual dysfunction, fatigue, dysphagia, and sleep disturbances [31,32]. Major risk factors include older age, pre-existing frailty, multisystem organ failure, prolonged immobilization, extended duration of mechanical ventilation, hyperglycemia, corticosteroid exposure, and use of neuromuscular blockade [27,33]. Physical impairment, which has been shown to persistent in up to 90% of survivors at 3 years often coexists with psychological disorders, compounding recovery challenges [34].

### 2.5. PICS-Psychological Disorders

The activation of inflammatory and neuroendocrine pathways in sepsis and critical illness disrupt the hypothalamic–pituitary–adrenal axis, alter neurotransmitter balance, and dysregulate sleep–wake and stress responses [35,36]. These pathophysiologic processes may predispose survivors to a range of psychological sequelae, including depression, anxiety, and post-traumatic stress disorder (PTSD) [37]. The reported prevalence of psychological disorders following sepsis varies widely but remains consistently high across studies. Anxiety affects approximately 32–46%, depression 29–40%, and PTSD 12–44% of survivors within the first year after ICU discharge [10,38,39,40,41]. A German cohort study found that 54.8% of sepsis survivors were diagnosed with a mental health impairment (MHI) within the first year [42]. Compounding this burden is an increased risk of self-injurious behavior (aHR 1.15) and suicidality (aHR 1.22) following ICU admission [43]. Risk factors for the psychological components of PICS include younger age, pre-existing mental health conditions, sleep disturbances during hospitalization, and respiratory failure requiring mechanical ventilation [44,45]. These disorders can persist for months to years after discharge, in one study PTSD affected up to 25% of ICU survivors for as long as 8 years after discharge, leading to reduced quality of life, impaired social reintegration, and increased caregiver strain [26].

### 2.6. PICS-Family and Financial Impact

Often considered the fourth PICS domain, PICS-Family (PICS-F) refers to the adverse psychological, physical, and social effects experienced by family members or caregivers following a loved one’s critical illness [8,46,47]. These effects are driven by sustained stress, sleep disruption, anticipatory grief, and moral distress related to surrogate decision-making and prolonged uncertainty [46]. Common manifestations include fatigue, cognitive slowing, and poor sleep quality [48,49]. The predominant psychological sequelae mirror those seen in patients with PICS—anxiety, depression, and PTSD—affecting up to 70% of family members [48,50]. Risk is highest for spouses, those experiencing poor communication with ICU staff, those with a prior history of mental illness, and when the patient experiences severe illness or death [26]. The burden can persist for years, especially when patients survive with disability or die during their ICU stay [26].

In addition to physical and psychological effects, socioeconomic challenges—described as the “fifth domain”, PICS-Financial—further compound family burden. Up to one-third of caregivers report loss of household income [51], and those experiencing anxiety or depression demonstrate reduced work productivity and increased absenteeism [52].

Having outlined the manifestations of post-intensive-care-syndrome, the following section focuses on preventive strategies implemented during the acute and recovery phases of sepsis that shape this trajectory of recovery.

### 2.7. Intervention and Prevention Using the A2F Bundle

**Figure 3:**
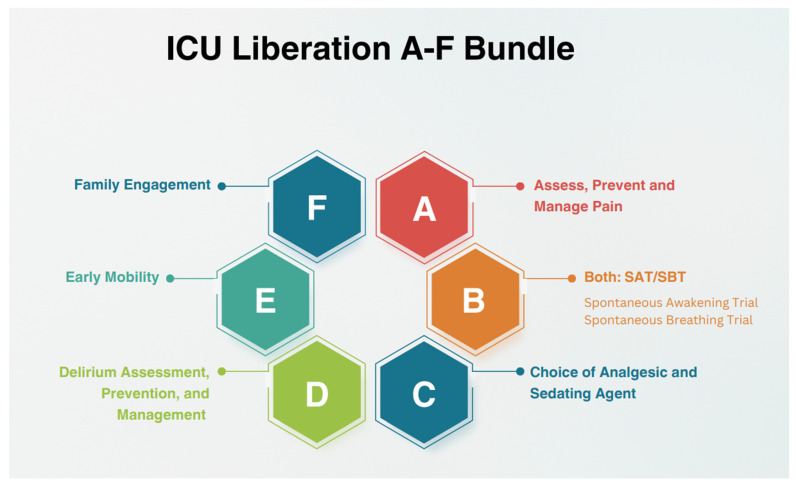
A-F ICU Liberation Bundle for Reduction in Post-Intensive Care Syndrome Morbidity.

A 2013 collaborative meeting produced updated ICU Pain, Agitation, and Delirium (ICU-PAD) guidelines and ultimately laid the foundation for the subsequent development of the ABCDEF bundle in 2018 [53,54]. The ABCDEF bundle (Figure 3) provides an evidence-based guide that offers clinicians and institutions opportunities to optimize ICU patient recovery and outcomes [53]. A recent 2024 multicenter, prospective cohort study found that implementation and adherence of the ABCDEF bundle in mechanically ventilated adult ICU patients led to reduced ICU length of stay, days of mechanical ventilation, and percentage of patients requiring more than 7 days in the ICU. These outcomes all represent targetable risk factors associated with PICS [55]. The following section highlights each component of the ABCDEF bundle and its application in the critically ill, septic shock population (Table 1).

#### 2.7.1. Assess, Prevent, and Treat Pain

Pain is a prevalent and clinically significant symptom that is often underreported or underassessed in ICU settings, particularly among sedated or mechanically ventilated patients [56]. It arises from multiple sources, including invasive catheters, underlying illness or surgery, and immobility. In communicative patients, the 1–10 numerical rating scale (NRS) is recommended for pain assessment, whereas the Behavioral Pain Scale (BPS) and the Critical-Care Pain Observation Tool (CPOT) are preferred for non-communicative patients [54,57]. The 2013 ICU-PAD guidelines advise analgesic medication for uncontrolled pain (defined as NRS > 4, BPS > 5, or CPOT > 3) and prior to procedures [53,54,57]. Whenever feasible, non-opioid medications and non-pharmacologic strategies—such as repositioning, heat or cold application, relaxation techniques, guided mindfulness—should be employed, though opioids remain first-line for non-neuropathic pain [58].

In sepsis and septic shock, systemic inflammation and altered cytokine signaling can alter nociceptive processing and patient’s pain thresholds. Poorly controlled pain during critical illness is linked to physiologic stress response, delayed recovery, and long-term mental health consequences [59]. A 2024 meta-analysis evaluating pain and delirium in older hospitalized adults demonstrated delirium to be twice as likely to occur with pain at rest and three times more likely with severe pain [60]. Inadequately treated pain also contributes to anxiety, depression, poor sleep, and demoralization, and both patients and families identify uncontrolled pain to be some of the most distressing aspects of ICU care [61]. Therefore, pain in patients with sepsis and septic shock should be systematically assessed and promptly treated to minimize complications and promote recovery.

#### 2.7.2. Both Spontaneous Awakening Trials (SAT) and Spontaneous Breathing Trials (SBT)

Mechanical ventilation is required in substantial proportion of patients with sepsis and septic shock, with rates ranging from 13 to 40%, with rates higher in patients with higher severity of illness [62,63]. These patients often receive continuous sedation to facilitate comfort and ventilator synchrony, which can inadvertently contribute to prolonged ventilation and delayed recovery.

Spontaneous awakening trials (SAT) involve the temporary cessation of all sedatives and analgesics to allow increased patient arousal while monitoring for evidence of uncontrolled, acute pain. Deep sedation in the first 48 h of ICU admission is associated with prolonged mechanical ventilation times, increased need for tracheostomy, and increased hospital and long-term death [64,65,66]. Conversely, SATs are associated with a 2-day reduction in days of mechanical ventilation and a 3.5-day reduction in ICU length of stay [67].

Spontaneous breathing trials (SBT) represent the reduction in ventilator support to assess patients’ ability to breathe spontaneously. SBTs are frequently implemented in ventilator weaning protocols and reduce the duration of mechanical ventilation [68,69]. When paired together, SAT and SBT improve patient outcomes. A multicenter, randomized controlled study demonstrated a 3.1-day reduction in mechanical ventilation and 3.8-day shorter ICU stay among patients receiving paired SAT and SBT compared to SBT alone [70].

Although data specific to patients with sepsis and septic shock are limited, early coordination of SAT and SBT in this population should be prioritized once hemodynamic stability is achieved. Implementing these protocols can shorten duration of ventilation, decreased ICU length of stay, and potentially mitigate the long-term sequelae of PICS.

#### 2.7.3. Choice of Analgesia and Sedation

Psychoactive medications are frequently required in the ICU, yet excessive sedation remains a major barrier to extubation and an independent risk factor for delirium. Reliable assessment tools are essential to target light sedation, balancing comfort with alertness. The Richmond-Agitation Sedation Scale (RASS), a 10-level numerical scale, has demonstrated high interrater reliability across many ICU settings. In a retrospective study of patients with sepsis, lower RASS scores—reflecting deeper sedation—were independently associated with worse prognosis and highly mortality [71]. The Riker Sedation-Agitation Scale serves as another validated alternative [72]. Sedation depth should be individualized, with frequent reassessment to maintain ligh sedation targets (RASS 0 to −1) whenever clinically feasible.

Once appropriate monitoring is in place, the next priority is selecting sedative and analgesic agents that minimize adverse cognitive outcomes. Commonly used medications include propofol, dexmedetomidine, midazolam, lorazepam, fentanyl, hydromorphone, morphine, and ketamine [73]. In septic patients, early studies comparing dexmedetomidine with benzodiazepine-based sedation demonstrated significantly lower rates of delirium and coma, with favorable trends toward shorter ventilation duration and improved 28-day survival [74]. These findings reinforced the association between benzodiazepine exposure and increased risk of delirium [75,76]. More recently, a study comparing dexmedetomidine and propofol reported similar outcomes in delirium, ventilator-free days, mortality, and long-term cognitive impairment [77].

For patients with sepsis and septic shock requiring mechanical ventilation, sedation should be carefully titrated to the lightest effective level. Preference for non-benzodiazepine agents—such as propofol or dexmedetomidine—offers the potential to reduce delirium incidence, shorten ventilation duration, and improve long-term cognitive impairment associated with PICS.

#### 2.7.4. Delirium: Assess, Prevent, and Manage

Delirium is a major concern in the ICU and is a strong predictor of prolonged cognitive dysfunction following critical illness. It occurs in approximately 50–75% of mechanically ventilated patients [58] and ranges from 24 to 76% in patients with sepsis and septic shock in the ICU [78,79]. The Confusion Assessment Method for the Intensive Care Unit (CAM-ICU) remains the most widely validated tool for identifying delirium and is based on four core features: acute change in mental status, inattention, altered level of consciousness, and disorganized thinking. A positive CAM-ICU requires both acute altered mentation and inattention with either altered level of consciousness or disorganized thinking and has a 95.9% specificity for delirium. When combined with the RASS, the CAM-ICU may be used to identify subtypes of delirium [80,81,82]. Hyperactive delirium (CAM-ICU positive, RASS ≥ 1) is characterized by agitation, restlessness and emotional lability [80], whereas hypoactive delirium (CAM-ICU positive, RASS ≤ 0) presents as withdrawal and decreased arousal. The latter is more common in the critically ill and was accounted for 89% of delirious patients in the 2018 MIND-USA trial [70,82]; though less frequent, hyperactive delirium, is associated with a more favorable prognosis [80].

Prevention of delirium in septic patients requires both optimal sepsis resuscitation and mitigation of delirium-provoking factors such as uncontrolled pain, oversedation, and sleep disruption [10,25,83]. Effective management emphasizes proactive prevention through light sedation, avoidance of anticholinergic and deliriogenic medications, promotion of normal sleep–wake cycles, and early mobilization. While antipsychotics have historically been used for treatment, evidence does not support routine pharmacologic therapy. The MIND-USA study found no difference in delirium duration between haloperidol, ziprasidone, and placebo [84], and the AID-ICU trial similarly demonstrated no improvement in mortality, delirium duration, or days alive and out of the hospital with haloperidol use [85]. Quetiapine has shown modest benefit in hyperactive delirium, with reduced delirium severity, shorter ICU stays, and improved sleep quality compared to haloperidol, thought without a demonstrated survival benefit [86]. Beyond its sedative role, dexmedetomidine has also shown potential therapeutic benefit in the management of established ICU delirium, shortening delirium duration and increasing ventilator-free days compared to haloperidol or placebo, albeit at the cost of increased rates of bradycardia [87].

Therefore, in patients with sepsis and septic shock, daily delirium screening with validated tools such as the CAM-ICU, combined with proactive prevention—including light sedation, sleep preservation, and early mobilization—should be standard practice to reduce delirium-related morbidity and long-term cognitive impairment.

#### 2.7.5. Early Mobility and Exercise

Early critical care practices traditionally emphasized deep sedation and immobilization [69]. Over time, mounting evidence has shown that prolonged immobility contributes to loss of lean muscle mass, longer hospitalizations, and persistent functional decline that can last up to five years after discharge [88,89,90]. The inflammatory and catabolic state of sepsis and septic shock further accelerates muscle breakdown, amplifying the risk of ICU-acquired weakness and subsequent physical impairment [29].

Understandably, clinicians may hesitate to pursue early physical therapy and ambulation in patients receiving invasive therapies such as mechanical ventilation, continuous renal replacement therapy, and extracorporeal oxygenation. However, evidence supports the feasibility of collaborative mobilization efforts in these complex patients [91,92]. Early initiation of physical rehabilitation—particularly within the first three days of ICU admission—was associated with improved survival, shorter duration of delirium, and greater likelihood of regaining functional independence at discharge [93,94]. In sepsis-specific cohorts, early mobilization (within 3 days) significantly reduced ICU-acquired weakness at hospital discharge [95]

Despite these proven benefits, establishing a sustainable ICU rehabilitation program remains challenging, requiring engaged multidisciplinary champions, adequate staffing, and institutional support [96]. Nevertheless, integrating early mobility into sepsis care provides a critical opportunity to mitigate ICU-acquired weakness, preserve cognition, and reduce the physical sequelae of post-intensive-care syndrome. Accordingly, in patients with sepsis and septic shock, early and progressive mobilization should be prioritized to improve functional outcomes and reduce long-term disability.

#### 2.7.6. Family Engagement and Empowerment

In patients with sepsis and septic shock, the acute and unpredictable course of illness places tremendous emotional and decisional strain on families, making structured engagement a vital component of comprehensive care. The impact of critical illness can be as profound for families as for patients themselves. ICU-related factors to PICS-F include poor communication, limited visiting hours, and lack of participation in decision-making [97,98]. A 2018 trial demonstrated that a multicomponent family support intervention—including prescheduled nurse-meetings—improved communication, enhanced understanding of the patient’s condition, and reduced ICU length of stay [99,100]. Similarly, ICU diaries have been shown to help families process the patient’s trajectory, foster emotional recovery, and improve post-discharge adjustment [101].

Ineffective or unclear communication, particularly around prognosis and treatment decisions, is strongly associated with higher family anxiety and depression [102,103]. In contrast, proactive engagement fosters shared understanding, supports value-concordant care, and mitigates caregiver distress. The 2024 Society of Critical Care Medicine (SCCM) Family Guidelines recommend including families on daily rounds and liberalizing visitation policies to enhance involvement and transparency [104].

In summary, family engagement and empowerment should be integral to sepsis and septic shock management. Actively involving families in communication, decision-making, and care planning supports emotional recovery and aligns with evidence linking comprehensive ABCDEF bundle use to improved short- and long-term outcomes [105].

### 2.8. Rehabilitation and Future Directions

Post-Intensive Care Syndrome Recovery Clinics (PICS-RC), first established in 2012, were modeled after the post-critical care clinics pioneered in the United Kingdom [106,107]. These multidisciplinary programs typically integrate physicians, nurses, pharmacists, behavioral health specialists, and physical or occupational therapists to address the complex constellation of cognitive, physical, and psychological impairments affecting ICU survivors and their families [107].

Recognizing the high prevalence and multidimensional burden of PICS, the Society of Critical Care Medicine (SCCM) recommends that patients at elevated risk be screened for post-ICU impairments within 2–4 weeks after discharge and, when feasible, be referred for follow-up in a PICS-RC clinic within 6–12 weeks [108,109]. The 2021 Surviving Sepsis Guidelines further support this approach, advocating that all survivors of sepsis and septic shock undergo comprehensive evaluation of physical, cognitive, and psychological domains following hospitalization [17].

During PICS-RC visits, standardized assessments such as the Montreal Cognitive Assessment (MOCA), Hospital Anxiety and Depression Scale (HADS), Impact of Event Scale-Revised (IES-R), and a 6 min walk test are commonly employed to quantify deficits and guide individualized rehabilitation plans [108,109]. Early identification of impairment allows for timely interventions, potentially reducing long-term disability and caregiver burden.

Evidence supporting PICS-RC remains mixed. A randomized pilot study of an ICU recovery program suggested a longer time to hospital readmission compared to usual care, thought it was underpowered for clinical endpoints [110]. A larger pragmatic RCT using a coordinator-led mobile, nurse-led recovery program in survivors of respiratory failure did not show significant improvement in 12-month outcomes, including QOL and mortality [111]. Observational cohorts consistently report high rates of detecting cognitive, psychological, and functional impairments with frequent modifications to treatment plans at follow-up, thus supporting the role of clinics in coordinating rehabilitation [112]. While PICS-RCs are feasible and can improve processes of care, the quality of evidence for improvement in mortality, readmissions, or health-related QOL remains low due to heterogeneity in study methods and small study sizes—underscoring the need for larger, multicenter trials.

Ongoing research dedicated to PICS will be critical to improving outcomes for patients with sepsis and septic shock. Leveraging large-scale electronic health record data through networks such as the Critical and Acute Illness Recovery Organization (CAIRO) may enable development of predictive models to identify sepsis survivors most vulnerable to PICS—facilitating early intervention, personalized post-ICU care pathways, and more efficient resource allocation. In parallel, optimizing analgesic and sedative strategies remains central to preventing delirium—a key mediator of long-term cognitive dysfunction. Emerging data suggest that dexmedetomidine, increasingly utilized in septic shock, may confer benefits beyond sedation, including reduced vasopressor requirements and potential survival advantages [113,114]. Clarifying its mechanistic effects on catecholamine responsiveness, neuroinflammation, and brain recovery represents an essential next step in understanding how early ICU management influences long-term survivorship outcomes.

Looking ahead, research should prioritize not only the pathophysiologic mechanisms of PICS but also implementation strategies to enhance adherence to the ABCDEF bundle and strengthen post-ICU follow-up care. Large-scale multicenter initiatives, such as SCCM’s ICU Liberation Collaborative, demonstrate that sustained compliance requires institutional investment, workflow integration, and multidisciplinary engagement. Moreover, health systems research is needed to examine how disparities in socioeconomic status, health literacy, and access to rehabilitation influence PICS outcomes. Incorporating telehealth-based follow-up and digital monitoring tools into PICS recovery models may extend the reach of care to underserved populations.

## 3. Conclusions

Advances in early recognition and aggressive resuscitation have markedly improved survival among patients with sepsis and septic shock. Yet this success has introduced a new imperative: addressing the growing burden of post-treatment complications. As the prevalence and impact of post-intensive care syndrome (PICS) become increasingly recognized, the focus of critical care must extend beyond immediate survival toward optimizing recovery and long-term function.

Framing sepsis survivorship as a continuum—from acute resuscitation through long-term rehabilitation—underscores that the quality of recovery is determined by decisions made early in critical illness. The ABCDEF bundle provides a practical framework to bridge this continuum, reducing the incidence and severity of PICS while promoting meaningful recovery.

Ultimately, sepsis and septic shock should be viewed not only as acute medical emergencies but as sentinel events for enduring physical, cognitive, and psychological sequelae. By integrating early rehabilitation, structured follow-up through PICS recovery clinics, and ongoing support for patients and families, critical care professionals can redefine success in sepsis—not merely by survival, but by restoration of function and life participation.

## Figures and Tables

**Table 1:** ABCDEF Bundle Assessment Tools, Interventions, and Benefits.

	Assess, Prevent, and Treat Pain	Both SAT and SBT	Choice of Analgesia and Sedation	Delirium: Assess, Prevent, and Manage	Early Mobility and Exercise	Family Engagement and Empowerment
**Assessment Tools**	Numerical Rating Scale (NRS); Behavioral Pain Scale (BPS); Critical-Care Pain Observation Tool (CPOT)	SAT and SBT Screening Protocols	Richmond Agitation–Sedation Scale (RASS); Riker Sedation–Agitation Scale	Confusion Assessment Method for the ICU (CAM-ICU); Intensive Care Delirium Screening Checklist (ICDSC)	Physical/occupational therapy screening tools; functional strength scales	Family satisfaction/engagement surveys; documentation of participation in rounds
**Intervention**	Routine pain assessment; pre-procedure analgesia; multimodal analgesia favoring non-opioid and non-pharmacologic adjuncts	Paired SAT and SBT	Light sedation targeting RASS 0 to –1; preference for non-benzodiazepine agents (propofol, dexmedetomidine)	Daily screening; minimize benzodiazepines; maintain sleep–wake cycles; early mobility	Progressive mobility within 72 h of stability; multidisciplinary rehab team	Structured family meetings, ICU diaries, open visitation, inclusion on rounds
**Short term Benefits**	Reduced agitation, stress response, and physiologic instability	Shorter ventilation duration and ICU stay; decreased delirium	Less delirium, shorter ventilation and ICU stay	Reduced delirium duration, fewer days of coma	Reduced delirium, shorter ventilation and LOS	Improved communication, shorter LOS, lower family anxiety/depression
**Long term Benefits**	Lower risk of anxiety, depression, and chronic pain syndromes	Lower mortality, improved functional recovery, reduced PICS risk	Improved cognition and reduced long-term neurocognitive impairment	Improved cognitive outcomes, reduced long-term neuropsychiatric morbidity	Improved functional independence and quality of life; reduced PICS-related weakness	Reduced PICS-F burden, enhanced caregiver recovery, better long-term adherence to care goals

## Data Availability

All data can be found through literature search utilizing the methods detailed in the methods section of this manuscript. There are no potential conflicts of interest to disclose.
